# Adult Stem Cells and Diseases of Aging

**DOI:** 10.3390/jcm3010088

**Published:** 2014-01-21

**Authors:** Lisa B. Boyette, Rocky S. Tuan

**Affiliations:** 1 Center for Cellular and Molecular Engineering, Department of Orthopaedic Surgery, University of Pittsburgh, Pittsburgh, PA 15219, USA; E-Mail: lbb21@pitt.edu; 2 McGowan Institute for Regenerative Medicine, University of Pittsburgh, Pittsburgh, PA 15219, USA; 3 Department of Bioengineering, University of Pittsburgh, Pittsburgh, PA 15261, USA

**Keywords:** aging, stem cells, reprogramming, progeria, longevity, FoxO, Wnt, metabolic disease, oxidative stress

## Abstract

Preservation of adult stem cells pools is critical for maintaining tissue homeostasis into old age. Exhaustion of adult stem cell pools as a result of deranged metabolic signaling, premature senescence as a response to oncogenic insults to the somatic genome, and other causes contribute to tissue degeneration with age. Both progeria, an extreme example of early-onset aging, and heritable longevity have provided avenues to study regulation of the aging program and its impact on adult stem cell compartments. In this review, we discuss recent findings concerning the effects of aging on stem cells, contributions of stem cells to age-related pathologies, examples of signaling pathways at work in these processes, and lessons about cellular aging gleaned from the development and refinement of cellular reprogramming technologies. We highlight emerging therapeutic approaches to manipulation of key signaling pathways corrupting or exhausting adult stem cells, as well as other approaches targeted at maintaining robust stem cell pools to extend not only lifespan but healthspan.

## 1. Introduction

Diseases of aging constitute a huge burden for society, both in terms of economic cost and quality life-years of the population. The need for therapies to prevent and/or correct age-related molecular events leading to these diseases is growing. These include metabolic syndrome and diabetes, atherosclerosis, neurodegenerative diseases, osteoporosis, and cancer (Table 1, [1]). Onset of these diseases is highly correlated to advancing age across organ systems. Though molecular mechanisms contributing to cancer formation have been extensively studied, the mechanisms underlying age-related disease on the whole have only begun to be elucidated [2]. Molecular changes associated with age include telomere dysfunction, oxidative stress and deranged mitochondrial metabolism, inflammation, and cellular senescence, as well as altered signaling of sirtuins, insulin/insulin-like growth factor-1 (IGF-1), and the mammalian target of rapamycin (mTOR) pathway [3,4,5].

**Table 1:** Conserved aging phenotypes.

Phenotype	*H*. *sapiens*	*M*. *musculus*	*D*. *melanogaster*	C. *elegans*
Decreased cardiac function	Yes	Yes	Yes	NA
Apoptosis, senescence (somatic cells)	Yes	Yes	Yes	?
Cancer, hyperplasia	Yes	Yes	No	No
Genome instability	Yes	Yes	Yes	Yes
Macromolecular aggregates	Yes	Yes	Yes	Yes
Reduced memory and learning	Yes	Yes	Yes	NA
Decline in growth hormone (GH), dehydroepiandrosterone (DHEA), testosterone, IGF	Yes	Yes	?	?
Increase in gonadotropins, insulin	Yes	Yes	?	?
Decreased thyroid function	Yes	Yes	NA	NA
Decrease in innate immunity	Yes	Yes	Yes	Yes
Increase in inflammation	Yes	Yes	No	No
Skin/Cuticle morphology changes	Yes	Yes	?	Yes
Decreased mitochondrial function	Yes	Yes	Yes	Yes
Sarcopenia	Yes	Yes	Yes	Yes
Osteoporosis	Yes	Yes	NA	NA
Abnormal sleep/rest patterns	Yes	Yes	Yes	?
Decrease in vision	Yes	Yes	?	NA
Demyelination	Yes	Yes	?	No
Decreased fitness	Yes	Yes	Yes	Yes
Arteriosclerosis	Yes	No	NA	NA
Changes in fat	Yes	Yes	?	?

Reprinted by permission from Macmillan Publishers Ltd.: *Nature* [1], copyright 2008.

A three-pronged approach exists to combatting diseases of aging in the clinic, and further research can improve all three areas. The first arm is prevention of age-related disease through better understanding of the molecular causes of systemic aging and age-related disease. The second is pharmacologic intervention to reverse, correct, or prevent age-related disease. The third, in lieu of therapies that prevent and correct age-related molecular changes, is to surgically repair degenerated tissues. This includes engineering cells and tissues *ex vivo* to replace or augment regeneration of those in diseased and injured solid organs, nervous system components, and musculoskeletal structures [6,7,8,9,10]. The use of adult stem cells for this third approach, as well as the suspected regenerative functions of adult stem cells *in vivo*, has led researchers to closely consider the effects of systemic aging on the stem cell pools of an individual. 

Stem or progenitor cells are generally considered highly promising candidate cells for regenerative applications not only because they possess a high proliferative capacity and the potential to differentiate into other cell types [11,12,13,14], but also because they can be sourced autologously, eliminating any concerns about rejection or need for immunosuppressive therapy [8,15,16,17,18,19]. They possess innate immunomodulatory properties, home to sites of injury or inflammation, and direct the cells around them to begin a repair process via the production of bioactive factors and signaling molecules. Stromal stem cells are currently in clinical use as gene delivery agents to enhance tissue regeneration, to destroy cancer cells, and to regenerate cartilage and bone, and hematopoietic stem cells (HSCs) have been in clinical use for many years to reconstitute the immune system in cancer and other illnesses. However, studies of adult stem cells show that they do not fully retain their proliferative and multi-lineage differentiative capabilities in aging humans or after prolonged *ex vivo* propagation. Genetically or epigenetically modifying adult stem cells either to rejuvenate those of an elderly individual or to confer resistance to cellular aging during *ex vivo* propagation would yield a favorable cell source for regenerative medicine applications. Understanding the events that contribute to stem cell aging and developing methods to reverse those changes will also facilitate development of therapies to maintain *in vivo* adult stem cell pools as people age.

## 2. Adults Stem Cells and Causes of Aging

Adult stem cells are thought to reside *in vivo* as self-renewing pools and facilitate repair/replacement of damaged tissues over the lifespan of the organism. Stem cell quiescence lies on one end of a spectrum of self-renewal potential spanning from quiescence, to robust proliferation, to senescence, and death. Maintaining stem cell quiescence is essential for preserving the long-term self-renewal potential of the stem cell pool in a number of organ systems, such as the brain, bone marrow, musculoskeletal system, and skin [20,21]. There is an emerging body of evidence that altered and decreased function of adult stem cells *in vivo* secondary to accumulated metabolic stress plays an important role in the initiation of diseases of aging [22,23]. This is true in multiple organ systems. For example, in bone studies on the osteoblastic *versus* osteoclastic differentiation of progenitors in aging mouse models have shown that, over time, osteoblastic potential of stromal progenitors decreases, while osteoclastic differentiation of hematopoietic progenitors increases. This suggests an organismal aging program that results in common diseases of aging, including decreased bone quality [24]. Another example is in the immune system, where clonal diseases of myeloid stem cells occur more frequently and become more resistant to therapy with increasing age [25]. The hypothesis is now being investigated that this is caused by age-related genomic instability, causing a defective DNA damage response that results in abnormal differentiation of HSCs (reviewed in [26]). 

### 2.1. Self-Renewal and Maintenance of Stem Cell Pools

It would appear that the primary hit to adult stem cells during aging is to their proliferative/self-renewal potential more than their ability to undergo terminal differentiation effectively, although this is somewhat lineage-dependent. HSC populations in mice have been shown to actually increase in number and frequency with age, but with reduced ability to divide, delayed cell cycle progression, and age-related genetic changes in cell cycle regulators such as p21 and p18 [27]. In humans lower numbers of neuronal progenitor cells have been found in aged brains compared to young brains, but this population is still responsive and proliferates in response to ischemic injury [28]. Circulating hematopoietic progenitors were shown to increase more dramatically in younger patients after cardiopulmonary bypass graft than in older patients, and advanced age was associated with impaired coronary microvascular response to vascular endothelial growth factor (VEGF) [29]. Conversely, advanced age has been associated with a higher S-phase fraction of circulating HSCs in patients with aplastic anemia, but this predisposed them to dysplasia and conversion to acute myeloid leukemia, indicative of abnormal HSC function [30]. Studies of adult stem cell isolation yield in elderly individuals have shown that equivalent numbers of adipose-derived mesenchymal stem cells (MSCs) can be isolated from older individuals undergoing vascular surgical procedures as from young, healthy individuals [31,32]. 

The question remains whether those cells can be adequately expanded in tissue culture and whether they are able to mobilize, proliferate, and effect tissue repair *in vivo* when they are needed. In fact these same studies [31,32] have shown that while advanced age does not impact availability of stem cells, fat from patients with diabetes yielded significantly fewer stem cells than fat from non-diabetic patients. This would suggest that stem cells in the context of aging should probably be considered distinctly from stem cells in the context of diseases of aging. Advanced glycation endproducts, which accumulate in the setting of advanced age or diabetes, have been shown to directly impair HSC function and induce MSC apoptosis [33,34].

Other studies have shown that patients of increasing age yield adipose-derived MSCs that can be differentiated. However, frequently these studies do not compare the quality of differentiation to that achieved with cells from younger patients, leaving the question of whether differentiation potential has declined unanswered [35]. In one study looking at the efficacy of MSC transplantation following myocardial infarction, cells from aged donors did not perform as well as cells from younger donors [36]. Similarly, MSCs obtained from young individuals have been induced to undergo neuroectodermal differentiation *in vitro*, but this effect cannot be replicated in MSCs from elderly individuals [37]. A study demonstrating that lineage fate of MSCs from human donors was unaffected by donor age also found that activation from quiescence, including replicative function and quality of differentiation, was negatively impacted by advanced donor age [38]. 

### 2.2. An Aging Immune Milieu

A further complication in teasing apart the effects of aging on adult stem cell compartments is the changing interaction between stem cell types and with an aging immune system (reviewed in [39]). For example, the health and age of marrow-derived stromal stem cells has been shown to have an impact on the quality of HSCs, both *in vivo* and upon co-culture *ex vivo* [40,41]. Chronic pro-inflammatory cues, such as circulating inflammatory cytokines, which are upregulated in aged individuals [42], may both dysregulate the differentiation of stromal cells, and in turn negatively impact their ability to support hematopoietic progenitors, resulting in further dysregulation of the immune compartment. Mouse models of premature aging have demonstrated induction of NF-κB signaling and secretion of high levels of pro-inflammatory cytokines as a causative factor in the accelerated aging phenotype [43].

### 2.3. Genomic and Transcriptomic Data

Network analyses of signaling pathways differentially regulated in aging suggest that, rather than being a tightly regulated, well-defined program, aging may reflect a destabilization of other programs over time. On the other hand, extreme differences in lifespan between evolutionarily closely related species would argue that there is a dominant central aging program that determines organismal lifespan. Research indicating that survival to old age is not correlated with absence of risk alleles for common age-related diseases, such as cancer, coronary artery disease, and type 2 diabetes also supports the idea of a prevailing aging program [44]. 

Similarly, gene expression profiling has been done in adult stem cells to examine the effects of age in the setting of osteoarthritis and vice versa, revealing that different sets of genes were differentially regulated in association with either aging or osteoarthritis [45,46]. The pathways associated with aging were closely associated with glycan metabolism, in contrast to osteoarthritis, which was heavily associated with aberrations in immune signaling genes and regulators of self-renewal and differentiation, such as Wnt-related transcripts. Another gene expression profiling study looking specifically in human skin showed sex-specific age-related changes, with females displaying increased expression of pro-inflammatory genes that was not observed in males [47]. A recent meta-analysis of genome-wide association studies performed to identify polymorphisms associated with diseases of aging revealed that genes associated with multiple diseases known to occur in elderly individuals are generally associated with pathways regulating either inflammation or cell senescence, with the most highly significant susceptibility locations mapping to regulators of senescence, leading the authors to conclude that germline genetic heterogeneity in regulators of these pathways contributes significantly to onset of age-related disease [3].

### 2.4. Heritable Longevity

Some of the most pertinent research to understanding the molecular mechanisms underlying aging, rather than the molecular effects resulting from aging, is in the area of heritable longevity and premature aging in humans. Many genetic variants, the value of which is unknown, have been identified in areas associated with longevity and disease resistance, including dietary restriction, metabolism, autophagy, stem cell activation, tumor suppression, DNA methylation, progeroid diseases, stress response, and neural processes [44,48]. One of these variants, a single nucleotide polymorphism (SNP) in the gene *TOMM40*, was found not to be directly linked to decreased longevity, but instead reflects a linkage disequilibrium with multiple isoforms of the *APOE* gene that are deleterious to longevity and have been strongly associated with elevated cholesterol, cardiovascular disease, Alzheimer’s disease, and cognitive decline and dementia, as well as serum IGF-1 levels in women [49]. 

Several genetic variants in the insulin/IGF-1 pathway have been associated with longevity or increased healthspan and include multiple SNPs from nine different genes along this signaling axis: *AKT1*, *FOXO1A*, *FOXO3A*, *GHR*, *GHRHR*, *IGF1R*, *IGFBP3*, *IGFBP4*, and *PTEN*. Indeed, common SNPs in *AKT1* and *FOXO3A* have consistently been associated with longevity in three independent cohorts [50], as well as a SNP in the *CAMKIV* gene, which *in vitro* has been shown to activate *AKT*, *SIRT1*, and *FOXO3A* [44]. A SNP in the *MNPP1* gene, which codes for an enzyme similar to phosphatase and tensin homolog (PTEN) that regulates intracellular levels of polyphosphates, critical for determining the rate of cell proliferation, has also been associated with longevity in meta-analyses of large-scale genome-wide association studies.

### 2.5. Premature Aging

Progeria, or premature aging, reflects an opposite outcome from long lifespan or long healthspan. Individuals with Hutchinson-Gilford progeria syndrome (HGPS), caused by a point mutation in *LMNA*, the gene for the lamin A nuclear envelope protein, experience premature aging as a result of nuclear defects that lead to impaired cell division and transcriptional deregulation (reviewed in [51]). This point mutation activates a cryptic splice donor site, leading to production of a dominant negative form of the lamin A protein which has been named progerin; this splice variant is also expressed at low levels in normal individuals, accumulates in some cell types with normal aging, and is expressed at higher levels in several human cancer cell lines [52]. Similarly, individuals with Werner’s syndrome, who display adult-onset progeria, have a defect in the WRNp protein, which is critical for DNA replication and repair. 

In both aging syndromes, telomere shortening and DNA damage synergistically destabilize the genome, leading to accelerated p53-dependent senescence and apoptosis; this phenotype has been rescued in experimental models by over-expression of hTERT or p53 inactivation [53,54,55], and this process has also been documented in normal human fibroblasts [56]. Increased rates of nuclear DNA damage in all cell types, in combination with impaired stem cell regeneration of damaged tissues, are thought to be directly responsible for the accelerated aging phenotype that is observed. As a result this disease has inspired the generation of several aging models, both transgenic animal models and *in vitro* systems employing induced pluripotent stem (iPS) cells derived from fibroblasts of HGPS patients [57,58,59,60,61,62,63,64]. From these models, it has been learned that high rates of cellular senescence and apoptosis due to increased nuclear DNA damage correlate very well with decreased lifespan, independent of increased rates of cancer, whereas models with comparatively low rates of cellular senescence and apoptosis display increased lifespan [55,65]. 

Comparison of tissue phenotypes observed in normal aging and HGPS suggests that lamin A may play an important role in sensing and transducing stress response signals critical for adult stem cell and niche maintenance in all individuals [66]. Progerin has been demonstrated to accumulate in MSCs, vascular smooth muscle cells, and fibroblasts, both in *in vitro* disease models and in human subjects, in association with disease and other signs of aging in the skin, musculoskeletal, and cardiovascular systems [59,67,68]. MSCs have been shown not only to be most susceptible to progerin accumulation and failed cell division, but also more susceptible to oxidative and other kinds of stress in the context of progerin accumulation both *in vitro* and *in vivo*. In the absence of normal lamin A or abundance of progerin, mild oxidative stress is sufficient to induce nuclear disorganization and premature senescence, confirming the importance of this protein for maintaining tolerance to reactive oxygen species [69]. These factors combine to effectively wipe out this adult stem cell pool in HPGS patients, leaving them with a critical deficit in tissue regeneration [59,70], and it is likely this same process plays a role in progressively declining MSC function with normal aging. 

### 2.6. *Ex vivo* Stem Cell Aging

A wholly separate but potentially instructive area of research involves those studies focused on *ex vivo* aging of adult stem cells. *Ex vivo* stem cell aging has been shown to be very similar to *in vivo* stem cell aging in rodent models, but this has not held true on a molecular level in every study done with human cells. For example, telomere shortening, which drives cellular senescence in cultured human cells, is not observed in rodent cells clearly undergoing replicative senescence [71]. Despite this finding, there does appear to be an association between *ex vivo* cell senescence and organismal lifespan, and studies of telomerase mutations in humans have revealed an association with diseases of aging in which tissue compartments require a high degree of cell self-renewal [72]. Similarly, short telomeres have been linked with some tissue-specific degenerative diseases, and telomere length is evaluated as a clinical parameter in determining therapeutic approaches (reviewed in [73]). Ablation of senescent cells in progeroid mice has been shown to delay or rescue the aging phenotype at the organismal level, implicating senescent cells in the pathogenesis of age-related disease *in vivo* [74]. Taken together, these findings suggest that the study of *ex vivo* senescence could yield information pertinent to *in vivo* aging. 

Examination of adult stem cell proliferation in *ex vivo* tissue culture have shown that MSC proliferation declines precipitously after repeated passaging [75]. Studies of differential gene expression between early and late passage MSCs showed progressive down-regulation of genes associated with self-renewal, such as *OCT4*, *SOX2*, and *TERT* and up-regulation of genes associated with osteogenic potential; this was accompanied by an increased propensity for spontaneous osteogenic differentiation and decreased proliferation over time [76]. The authors of this work noted a concomitant increase of epigenetic dysregulation of histone H3 acetylation in association with these differentially regulated genes, and correction of this dysregulation with fibroblast growth factor (FGF) administration during culture, resulting in promotion of proliferation and suppression of spontaneous osteogenesis.

## 3. Metabolic Stress and Adult Stem Cell Aging

Adult stem cells experience many stressful insults in the course of a lifetime of tissue repair. Regulation of energy metabolism is critical to withstanding stress, which comes in the form of nutrient deprivation, oxidative stress, DNA damage, pathogens, and other stressors. Studies on the molecular causes/effects of aging in adult stem cells have shown that in aged subjects these cells display an altered proteome, with proteins involved in cytoskeletal organization and anti-oxidant defense being age-dependent and associated with functional impairment of the cell, including decreased responsiveness to physical environmental cues and decreased resistance to oxidative stress [77]. 

### 3.1. Oxidative Stress

Mesenchymal stem cells (MSCs) from both bone marrow and adipose tissue have been shown to have reduced capacity for oxidative stress with increasing donor age [78,79,80]. Studies in patients undergoing percutaneous coronary intervention after myocardical infarction have shown that self-renewal capacity and therapeutic efficacy of autologous bone marrow-derived MSCs can be correlated with blood gas levels in the marrow niche. This indicates that the function of these cells is highly dependent on their redox status [81]. 

**Figure 1:**
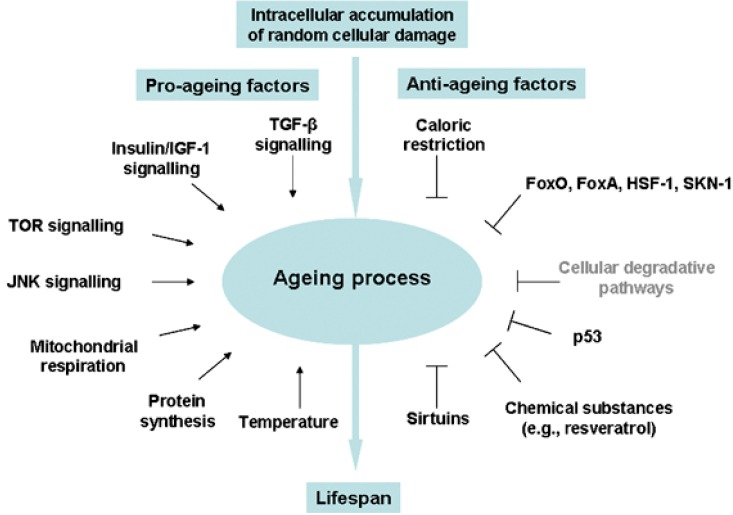
Environmental cues and evolutionarily conserved pathways that regulate the aging process in diverse eukaryotic phyla. Reprinted by permission from Macmillan Publishers Ltd.: *Cell Death and Differentiation* [86], copyright 2008.

Oxidative stress is increasingly being recognized as a fundamental underlying component of the aging process, leading to hyperactivity of pro-growth pathways like insulin/IGF-1 and mTOR, subsequent accumulation of toxic aggregates and cellular debris, and ultimately activation of cell death/survival pathways leading to apoptosis, necrosis, or autophagy (reviewed in [82]). Insulin/IGF-1, mTOR, FoxO, AMP-activated protein kinase (AMPK), and the sirtuin pathways all play a role in stem cell maintenance and differentiation through their sensing and regulation of energy availability in times of stress (reviewed in [83,84,85]), and these same pathways have been associated with advancing age in humans (Figure 1, [86]). Studies of two independent cohorts testing the expression of mTOR-related transcripts in aging found robust associations for genes involved in insulin signaling (*PTEN*, *PI3K*, *PDK1*), ribosomal biogenesis (*S6K*), lipid metabolism (*SREBF1*), cellular apoptosis (*SGK1*), angiogenesis (*VEGFB*), insulin production and sensitivity (*FOXO*), cellular stress response (*HIF1A*) and cytoskeletal remodeling (*PKC*), all of which were negatively correlated with advancing age, and for genes involved in inhibition of ribosomal components (4EBP1) and inflammatory mediators (STAT3), which were positively correlated with advancing age [87].

### 3.2. Stress Response and Homeostasis

In response to sublethal stress-induced insults, cells must remove or repair damaged components in order to reestablish homeostasis. Autophagy is one of the processes by which cells accomplish stress-induced metabolic adaption, and it has been identified as a critical mechanism for maintenance of stem cell function with aging [88]. Basal levels of autophagy are higher in adult stem cells from many tissue types compared to terminally differentiated cells, and autophagy is down-regulated during differentiation of adult stem cells. mTORC1, AMPK, and the sirtuins have all been shown to differentially regulate autophagy in response to nutrient stress, suggesting one possible connection between starvation and resistance to aging (reviewed in [89]). 

The sirtuins are a family of nicotinamide adenine dinucleotide (NAD^+^)-dependent deacetylases which are critical for maintaining cellular homeostasis in the face of age-related metabolic and other stressors, helping to prevent diseases of aging, but they are not thought to be involved in regulation of organismal lifespan (reviewed in [90]). Sirt1 coordinates stress responses and cell metabolism and regulates replicative senescence, is found in much higher levels in stem cells than differentiated cells, and is down-regulated upon differentiation of stem cells [91]. HSCs are dependent upon Sirt1 for maintenance of their undifferentiated state through elimination of reactive oxygen species, FoxO activation, and p53 inhibition [92]. In the case of embryonic development or tissue revascularization following ischemic injury, Sirt1 promotes endothelial progenitor branching and proliferation, although it is not required for endothelial cell differentiation [84]. These effects of Sirt1 are a result of its negative regulation of downstream effectors such as FoxO and Notch proteins. Resveratrol, a known Sirt1 agonist, has been shown to enhance osteogenic differentiation over adipogenic differentiation of MSCs, thereby conferring bone-protective effects and highlighting the importance of Sirt1 and its downstream target FoxO3 in preventing age-related osteoporosis [93]. Sirt1 confers sensitivity to insulin when over-expressed, and has been shown to be significantly down-regulated in cells resistant to insulin [94]. Given the critical function of this enzyme for maintaining robust adult stem cell pools and regulating their differentiation in multiple organ systems, its down-regulation in the context of insulin resistance provides one clue as to why metabolic disease is so damaging for regenerative processes in aging individuals.

## 4. Adult Stem Cells: Caught in the Balance between Cancer and Metabolic Disease

**Figure 2:**
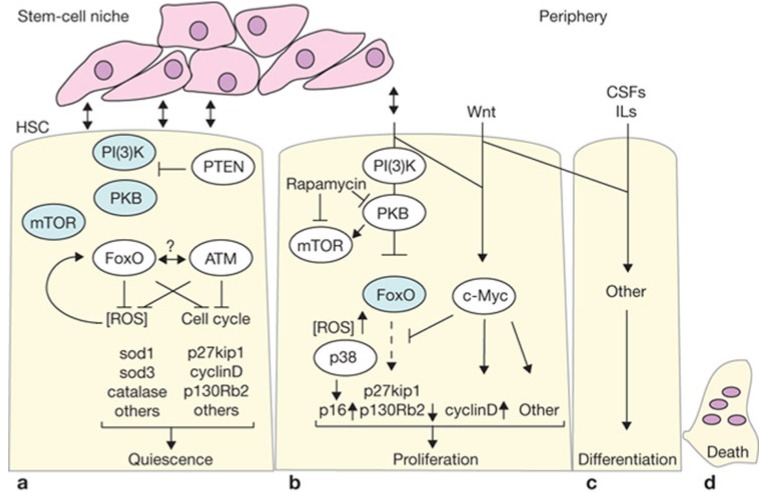
(**a**) PTEN is active in quiescent long term-HSCs and represses protein kinase B (PKB) signaling towards downstream components (such as mTOR and FoxO). Active FoxO programs cells to remain quiescent by cell-cycle repression and other mechanisms, but also allows survival by switching to a metabolic program of gluconeogenesis and fatty acid metabolism, together with elimination of reactive oxygen species (ROS). Ataxia telangiectasia mutated (ATM) may function in conjunction with FoxO through an as yet undefined mechanism, probably involving regulation of ATM expression by FoxO. Proteins active in LT-HSCs are shown in white elipses; (**b**) HSCs are driven to proliferate after loss of PTEN or FoxO. PKB and other downstream phosphoinositide 3-kinase (PI(3)K) events are active in this situation (indicated in white elipses). Loss of FoxO results in increased intracellular ROS levels, which in turn activates p38. If PI(3)K signaling is required to drive LT-HSCs into proliferation under normal conditions, what external niche signals would do so are unknown. However, PI(3)K signaling may function in cooperation with other signaling pathways, and this is illustrated by c-Myc—a downstream target of Wnt signaling. Myc represses FoxO, and may also independently regulate control of proliferation; (**c**) Differentiation of lineage-restricted cells further continues and is guided by extracellular signals such as interleukins (ILs) and various colony stimulating factor (CSFs); (**d**) After executing their function, all hematopoietic cells die and regeneration begins. Modified with permission from Macmillan Publishers Ltd.: *Nature Cell Biology* [97], copyright 2007.

Regulation of organismal longevity is coordinated through many intersecting signaling pathways that maintain a tight balance between carcinogenesis and apoptosis in individual cells [95,96]. For a stem cell, which over the course of its existence travels on the self-renewal spectrum from unlimited proliferative potential to senescence and ultimately death, many of these pathways are at work in opposition to each other all the time (Figure 2, [97]). A number of the well characterized pathways that control cell proliferation in cancer are now being examined for their role in regulating stem cell renewal and aging. One group took advantage of this similarity in regulatory networks between cancer cells and stem cells to study the effect of anti-aging reagents on induction and maintenance of self-renewal behavior and underlying mechanisms in stable cancer cell lines. They found that *BMI1*, a well-known proto-oncogene and critical regulator of self-renewal in multiple adult stem cells populations, took over the epigenetic program in cells retrogressing to a more primitive state as a result of the anti-aging treatments [98]. This proto-oncogene has also been identified as a critical promoter of osteogenesis through its coordinated stimulation of *SIRT1* expression and inhibition of p16, p19, and p27 in response to pararthyroid hormone related peptide (PTHrP) signaling [99], resulting in enhanced proliferation, decreased apoptosis, and decreased adipogenic differentiation of MSCs [100]. 

### 4.1. Proliferation *vs.* Oncogenic Resistance at the Cellular Level

Ultimately aging at the cellular level is suspected to result from a disruption of the balance between alternative cellular states (reviewed in [101]), with proto-oncogenes that promote stem cell function, such as *BMI1* and Wnt/β-catenin, operating in opposition to tumor suppressor genes that induce death or senescence in stem cells, such as *INK4A* (p16^Ink4a^) and *ARF* (p19^Arf^) [102]. In the hematopoietic system, aging has been closely linked with impaired repair response to DNA damage, leading to increased propensity for dysplastic syndromes and ultimately cancer [26]. The occurrence of cellular senescence, in contrast to quiescence or proliferation, is thought to be a protective response against oncogenic insults. Expression of *INK4A* has been shown to increase with age, and this progressive increase in tumor suppressor activity independent of levels of proto-oncogene expression may account for reduced stem cell activity with aging [102]. The tumor suppressor AIMP3/p18, endogenous levels of which increase in aged human tissues, drives cells to senescence when overexpressed. In transgenic mice p18 promotes a progeroid phenotype through selective degradation of normal lamin A [103]. 

A frequently raised concern about the therapeutic use of autologous adult stem cells from aged individuals is precisely that with the life-long accumulation of potentially mutagenic insults to their DNA, if they are still capable of robust self-renewal, they might pose an increased risk for cancer upon mobilization or exogenous activation [104]. That said, several studies have indicated that over the course of human development, as the need for growth decreases and the risk of oncogenesis increases, requirements for critical tumor suppressor mechanisms change, with adult stem cells displaying dependence on self-renewal regulatory signaling pathways that are not necessary in embryonic or even fetal stem cells [105].

### 4.2. Growth *versus* Oncogenic Resistance at the Organismal Level

Just like the constant struggle between carcinogenesis and apoptosis at the cellular level, growth *versus* somatic preservation is also balanced at the organismal level throughout life. One emerging hypothesis of aging is that it reflects tissue dysfunction due to hypertrophy and hyperplasia, rather than tissue damage [106], plus senescence resulting from prolonged hypertrophic arrest [107]. Insulin/IGF-1 signaling, turned on at the systemic level in response to glucose or growth hormone, is a potent stimulator of cell growth and proliferation via the Akt-TOR pathway, regulating organismal growth in childhood and anabolic metabolism in adulthood. As discussed above, gene variants along this pathway have been associated with longevity (age ≥92 years) in clinical cohorts [50]. Interestingly, reduced IGF-1 levels are present not only in extremely long-lived individuals, but also in progeroid individuals. This illustrates that suppression of this axis is not a causative factor in increasing or decreasing lifespan and healthspan, but rather an adaptive response against accumulating DNA damage at the expense of growth and other metabolically expensive processes [108,109,110]. Adult stem cells are highly responsive to insulin/IGF-1 signaling and programmed to replicate and repair; both metabolically costly activities. As somatic DNA is exposed to a lifetime of potentially mutagenic hits, these metabolically active cells are increasingly caught in the balance between cancer and metabolic disease, the hallmark of which is insulin resistance. 

### 4.3. Manipulation of Growth Pathways

Pharmacologic agents targeting the insulin/IGF-1 axis—both neutralizing monoclonal antibodies against IGF-1/IGF-1R and tyrosine kinase inhibitors which target the insulin receptor and IGF-1R—have been developed to treat cancer, but in clinical trials to evaluate these agents, a common side effect was hyperglycemia due to inhibition of insulin signaling [111]. Similarly, metformin, an agent already in use for the treatment of type 2 diabetes, has been shown to reduce the incidence of cancer, in part through AMPK-dependent inhibition of mTOR (thus cell growth) and AMPK-independent cell cycle arrest [112], but also in part through decreased levels of insulin and insulin resistance (reviewed in [113,114,115]). 

Manipulating this pathway to combat disease in aging humans is fraught with complications, and creative, highly specific approaches are required to avoid trading one disease for another. This is especially important to consider in the case of stem cells. Chemotherapy-resistant cancer stem cells, characterized by a high degree of metabolic flexibility, have been shown to be very sensitive to metformin [116,117]. One of the proposed mechanisms of action of metformin on cancer stem cells is interference with TGFβ-induced epithelial-to-mesenchymal transition [118], and metformin has been shown to prevent transcriptional activation of *OCT4* through AMPK activation [119]. These properties of metformin should generate concern about the effects on adult stem cell populations during its use for treatment of diabetes and cancer, but studies have also shown that while metformin-induced AMPK activation interferes with mechanisms critical for cancer stem-cell related tumorigenesis, adult stem cells may be less susceptible to disruption by metformin [120]. 

Accumulating evidence suggests that pathways governing self-renewal have distinct effects on normal stem cells and cancer stem cells even within the same tissue. In their study demonstrating adult stem cell dependence on PTEN in the hematopoietic compartment, in contrast to cancer stem cells, Yilmaz *et al.* [121] discussed several mechanisms through which maintenance of normal adult stem cells may be different from that of cancer stem cells. They suggested that persistent activation of PI(3)K in the absence of PTEN inhibition may lead to the accelerated exit of normal HSCs from the progenitor pool [121], and later showed that PTEN deficiency induces senescence and apoptosis in normal HSCs via increased expression of the cell cycle-regulating tumor suppressors p16 and p53 [122], in contrast to other cells from the hematopoietic compartment. Effects of PTEN deficiency on the HSC pool could be rescued by rapamycin, indicating events downstream of mTOR are responsible, for example changes in Akt signaling. It is known that mTOR inhibition activates FoxO signaling, resulting in increased stress resistance and longevity in invertebrate models [123]. It was hypothesized that prolonged rapamycin treatment might actually be inhibiting Akt signaling through mTORC2 rather than activating Akt through mTORC1, leading to loss of FoxO function, an attractive explanation for the accelerated stem cell aging observed with PTEN deficiency [124]. However, thus far this has not been shown to be the case in PTEN-deficient HSCs [105,122]. Cancer stem cells are able to escape this process through secondary mutations that attenuate mTOR-dependent tumor suppressive mechanisms [122]. 

Similar results have been obtained in HSCs with deletion of the cell cycle regulator p21, showing that control of cell cycle entry under conditions of stress is crucial for maintenance of stem cell quiescence and prevention of premature deletion of an entire adult stem cell pool [125]. Indeed, even in the case of pluripotent cells, metformin appears to have split effects: When administered to mice after iPS cell transplantation, metformin prevented teratoma formation but did not interfere with tissue formation from all three germ layers [126]. The findings are controversial regarding the effect of metformin on the differentiation of adult stem cells. In one study on rat marrow-derived MSCs, metformin enhanced osteogenesis at the expense of adipogenesis, presumably through modulation of peroxisome proliferator-activated receptor (PPAR)γ activity [127], opposite of the effect observed with glitazones, which activate PPARγ and can lead to bone loss [128]. However in another study of human and rabbit MSCs, metformin did not induce osteogenesis, while 5-aminoimidazole-4-carboxyamide ribonucleoside (AICAR), a small molecule activator of AMPK, induced robust osteogenic differentiation even in the absence of induction medium [129]. In studies of primary osteoblasts, activation of AMPK signaling was observed during early differentiation events, but chemical induction of AMPK with metformin blocked terminal differentiation and matrix mineralization [130]. 

### 4.4. Metabolic Dysregulation

Even in the absence of pharmacologic intervention targeting these pathways, metabolic disease is frequently the trade-off associated with oncogenic resistance. Perturbation of the aforementioned lifespan determinant pathways, such as SIRT1, insulin/IGF-1, FoxO and mTOR, leads to the development of metabolic syndrome features in mice [131]. Metabolic syndrome—characterized by the triumvirate of high cholesterol, high blood pressure, and high fasting blood glucose—and type 2 diabetes in turn lead to accelerated aging. In the case of full-blown type 2 diabetes, this accelerated aging is evidenced at the cellular level by slower DNA unwinding, increased collagen cross-linking, capillary basement membrane thickening, increased oxidative damage, and decreased Na^+^K^+^-ATPase activity, and at the organismal level by an increased incidence of cataracts, vascular disease and associated events (myocardial infarction, stroke, and pressure ulcers), cognitive decline, hip fracture, pain, incontinence, infections, and depression [132].

A vicious cycle then evolves whereupon metabolic disease can in turn indirectly increase oxidative stress and associated dysregulation of adult stem cell function [133]. An expanded adipose compartment produces higher levels of free radicals, leading to oxidative stress, one of the effects of which is to disrupt adipocytokine production. Adiponectin, an adipocytokine down-regulated in obesity and metabolic syndrome, is an important regulator of glucose and fatty acid metabolism, and in combination with other adipose-derived hormones, such as leptin, prevents insulin resistance (reviewed in [134]). One of the critical functions of adiponectin is to oppose the actions of angiotensin II, local (adipose) over-production of which also contributes to a pro-inflammatory state and increases oxidative stress *in vivo* (reviewed in [135]). 

The resultant disruption to homeostasis has many downstream effects that further increase inflammation and co-opt adult stem cells in worsening the situation. First, trafficking of multiple types of adult stem cells is likely altered in response to the inflammatory adipocytokines (IFNα, TNFα, IL-6) up-regulated during this process, leading some to speculate about adult stem cell exhaustion and the resulting impairment of tissue repair as the primary mechanism underlying long-term effects of metabolic disease, and in a less fulminant way the aging process in general [23]. Additionally, fate of adult stem cells is differentially regulated in this environment. For example, enhanced production in metabolic syndrome of 20-hydroxy-5,8,11,14-eicosatetraenoic acid (20-HETE) by the cytochrome P450 system and its cyclooxygenase-2-derived product 20-OH-PGE_2_ act to bias MSCs toward adipogenic differentiation through up-regulation of PPARγ and β-catenin, resulting in compounded inflammation-driven adipogenesis and impaired peripheral tissue maintenance through loss of otherwise uncommitted progenitors in patients with these disorders [136,137,138]. Activation of PPARγ has been shown to impair IGF-1 signaling in the marrow microenvironment [138], further contributing to skeletal loss, disruption of metabolic homeostasis, and potentially altering organismal lifespan [139]. In this way adult stem cells contribute to the pathogenesis of metabolic disease and also are impaired in their physiologic function by the presence of metabolic disease.

Despite the occurrence of insulin resistance and other attempts by cells to thwart oncogenic transformation in an aging metabolic system, metabolic disease is frequently associated with a higher incidence of cancers [140], particularly in sites with a high degree of cell metabolism and/or turnover. In part this reflects not a causal relationship but twin manifestations of a stressed system struggling and failing to restore homeostasis. However, the peripheral insulin resistance of metabolic disease also drives cancer growth through a decrease in hormone binding globulins (thus higher free steroid hormone levels), dysregulation of inflammatory cytokine and steroid and peptide hormone levels, and most importantly compensatory hyperinsulinemia, with many cancer cell types expressing high levels of insulin receptors and IGF receptors [141]. Large chromosomal clonal mosaic events, the incidence of which has been shown to increase with age [142], have been associated both with type 2 diabetes and with an increased risk of blood and solid organ cancers [143,144]. Clonal mosaicism in the blood compartment in particular further contributes to cancer formation, as well as increased susceptibility to disease in general, by leading to a reduced number of immune cell clones in circulation and resultant immunosenescence with age [142].

## 5.Illustrations of Deranged Signaling in Aging

### 5.1. Canonical Wnt Signaling: A Critical Pathway in Aging Stem Cells

As the MSC pool is skewed away from an osteogenic fate toward an adipogenic one in metabolic disease, there is massive disruption of signaling pathways that have implications for adult stem cells beyond differentiation. One of these pathways is the Wnt pathway, which is not only critical during development for axial patterning, but is also critical in stem cell fate determination. Many developmental events in stem cells are regulated by Wnt signaling, including self-renewal, differentiation, aging [145,146], and senescence [147,148,149]. Extensive crosstalk has been documented between Wnt signaling and FGF, prostaglandin E2 (PGE2), bone morphogenetic protein (BMP), Notch [150,151], TGF-β, and SMAD signaling pathways, with the common downstream target of this crosstalk being β-catenin [152,153]. In canonical Wnt signaling, β-catenin interacts with members of the T cell factor/lymphoid enhancing factor (TCF/LEF) transcription factor family to enhance expression of their target genes [146], which in turn regulate cell proliferation, carcinogenesis, differentiation, embryonic patterning, and stem cell maintenance [154,155,156]. 

The effects of Wnt signaling, however, have been shown to be highly tissue-specific and Wnt-specific. In the case of hair follicle stem cells in the skin, signaling through Wnt1 activated stem cell hyperproliferation via an mTOR-dependent mechanism, but long-term this activation of mTOR led to stem cell exhaustion and senescence [147]. This finding led the authors to conclude that while Wnt signaling can be a potent stimulus for stem cell proliferation, prolonged mTOR activation may serve as a protective mechanism to prevent tumor formation. The cost of this, of course, is exhaustion and depletion of that stem cell pool, ultimately resulting in impaired regeneration and aging of the tissue. In patients with acute myeloid leukemia, aberrant Wnt/β-catenin signaling, which controls self-renewal in the HSC pool, was higher in patients with unfavorable karyotypes and predicted a shortened survival [157].

In HGPS the tissue-specific patterns of accelerated aging point to a defect in MSC function. The defect in this adult stem cell compartment has been shown to be the result of both impaired self-renewal and dysregulated differentiation resulting from aberrant Notch and Wnt signaling [158]. Disruption of the nuclear lamina by progerin was shown to be directly responsible for downstream activation of Notch signaling effectors, spurring uncontrolled sporadic differentiation of MSCs along all three germ layers and enhanced osteogenesis at the expense of adipogenesis when differentiation was directed [159]. Wnt signaling was found to be severely disrupted in the progeroid *Zmpste24^−^*^/*−*^ mouse model (deletion of this enzyme causes restrictive dermopathy in humans), where the absence of normal lamin A resulted in an absence of active nuclear β-catenin in follicular stem cells, leading to down-regulation of cyclin D1 and repression of Akt and mTOR activation [160]. 

Further work has shown that *LEF1* is down-regulated as a result of this impaired nuclear translocation/retention of β-catenin; the absence of this transcription factor-activator complex in adult cells markedly reduced activation of canonical Wnt targets [161]. In this setting of severe Wnt inhibition, stem cells were not reduced in number, but instead entered a senescent state earlier and failed to proliferate, resulting in exhaustion of the functional stem cell compartment. The authors of this study also discovered increased apoptosis of the support cells in the stem cell microenvironment, which they suggested is another negative impact of defects in critical fate-determining signaling pathways that enable communication between tissue-resident stem cells and their niches.

Another mouse model of accelerated aging also points to aberrant Wnt signaling as a causative factor in degeneration due to stem cell defects, but from a different perspective. The Klotho mouse, which lacks klotho, a transmembrane and secreted β-glucuronidase involved in regulating insulin sensitivity among other functions, displays an accelerated aging phenotype, including short lifespan, infertility, arteriosclerosis, skin atrophy, osteoporosis, and emphysema [162]. Analysis of tissue-resident stem cells from multiple organs in Klotho mice revealed that they were reduced in number and displayed abundant senescence-associated markers prematurely [163]. Klotho was found to be a secreted Wnt antagonist capable of binding Wnts1, 3, 4, and 5a, and over-activation of Wnt signaling in Klotho mice drove tissue-resident stem cells into an early senescent phenotype, resulting in lack of self-renewal and stem cell compartment exhaustion. 

Taken together with findings regarding aberrant Wnt signaling in the *Zmpste24^−^*^/*−*^ mouse, these studies reveal the exquisite sensitivity of adult stem cell pools to the fate-determining effects of Wnt signaling, too much or too little of which results in failed maintenance of quiescent progenitors in adulthood. Perturbation of this pathway one way or the other has been demonstrated to result in adult stem cell aging and exhaustion, along with impaired differentiation, in the muscle compartment [164], the hematopoietic compartment [165,166], the vasculature [161], and the skeletal system [161,167] in addition to the skin, the gut, and the kidney. It is suspected that these processes unfold in normal aging as well as accelerated aging phenotypes, especially given the accumulation of low levels of progerin over time in normal individuals and declining serum levels of klotho in human aging [168].

### 5.2. The FoxO Family: Stem Cell Stress Response and Indirect Effects

**Figure 3:**
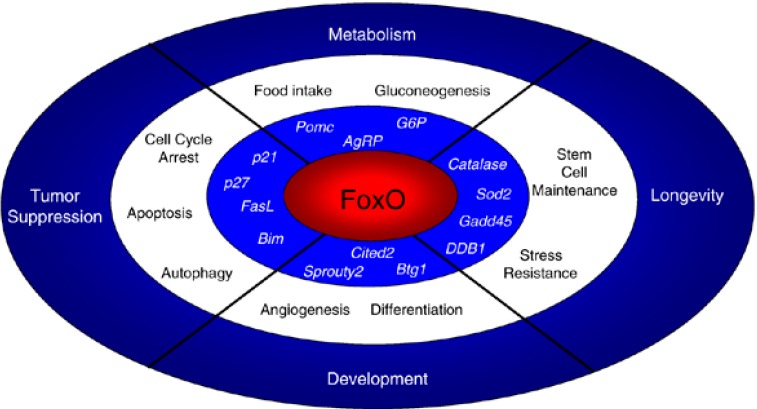
Roles of FoxO transcription factors in cells and in the organism. FoxO transcription factors trigger a variety of cellular processes by upregulating a series of target genes (in italics). The cellular responses elicited by FoxO affect a variety of organismal processes, including tumor suppression, longevity, development and metabolism. Reprinted by permission from Macmillan Publishers Ltd.: *Oncogene* [173], copyright 2008.

Another mechanism leading to altered Wnt signaling in aging is a shift in β-catenin binding to favor FoxO transcription factor signaling over the canonical Wnt pathway, mediated by TCF/LEF transcription factor signaling, in response to increasing oxidative stress. The FoxOs are a family of transcription factors regulating several of the previously discussed intersecting pathways, and have been shown to coordinate cell response in tumor suppression, metabolism, and organismal longevity (reviewed in [2,169,170]). In this capacity FoxO signaling acts to resist cellular stress opposite the growth-promoting signaling of mTOR [171]. FoxO transcription factors are downstream targets of insulin, growth factors, and nutrient and oxidative stress stimuli and in turn regulate several fundamental processes, depending on the cell type, including gluconeogenesis, neuropeptide secretion, cell cycle arrest, atrophy, autophagy, apoptosis, and stress resistance [169,172,173] (Figure 3, [173]). Furthermore, the FoxOs are an interesting family of transcription factors in that several are ubiquitously expressed, but display both highly specialized and universal functions in distinct cell types, as well as distinct and redundant functions which can be attributed to different FoxOs within the same cell type [2].

Studies on the effect of oxidative stress on adult stem cells suggest that it drives a shift in binding of the cellular β-catenin pool from the TCF/LEF family to the FoxO family [2,145,174]. As a cofactor in FoxO-mediated transcription, β-catenin facilitates defense against oxidative and other cellular stresses [175,176,177]. Diversion of β-catenin binding from TCF/LEF to FoxO, combined with inhibitory feedback to β-catenin/TCF signaling, serves to diminish canonical Wnt signaling [145,146,178,179]. This redirection of β-catenin function secondary to oxidative stress and resulting change in the cell’s transcriptional program have been implicated in several aging-related disease processes and cell senescence [97,146,149]. This transcriptional shift is particularly important in osteoporosis, where canonical Wnt signaling in osteoblasts is critical for skeletal homeostasis. This is one example of how increased activation of FoxO signaling by oxidative stress accumulated with age serves to directly undermine maintenance of an entire organ system, independent of the more broadly discussed “oxidative hypothesis of senescence”, wherein reactive oxygen species drive cells into arrest by activating FoxO signaling.

FoxO1 is of particular importance in age-associated skeletal disease. Osteoporosis, for example, is characterized by decreasing bone mass, which is attributed both to declining numbers of osteoblasts and declining function of osteoblasts. FoxO1 is critical for regulating osteoblast proliferation in the face of age-related oxidative stress and declining resistance to the effects of oxidative stress, both through its regulation of protein synthesis via interaction with the osteoblast-specific transcription factor ATF4 and through suppression of stress-induced p53 signaling which would otherwise lead to cell cycle arrest [180].

Several FoxOs regulate organismal metabolic function and thus play a role in metabolic diseases of aging and resistance to those diseases. FoxO1, through its regulation of osteocalcin secretion by osteoblasts, impacts pancreatic β-cell proliferation, insulin secretion, and insulin sensitivity [181]. In the case of atherosclerosis and vascular diseases of aging, FoxOs are important downstream effectors of PI(3)K, AMPK, and c-Jun N-terminal kinase (JNK) signaling. In the endothelium FoxO1 serves as a negative regulator of angiogenic behavior; this suppressive function, which is enhanced by activities of FoxO3 and FoxO4, is critical for organized vessel growth during development and repair [84]. FoxO1, FoxO3, and FoxO4 are also required to maintain endothelial quiescence within healthy vessels, and formation and remodeling of the endothelial barrier function is regulated by FoxO1 in a β-catenin-dependent manner.

FoxO transcription factors also play a role in attenuating diseases of aging through their regulation of the immune system and its progenitor pools (reviewed in [182]). In general, FoxOs regulate survival, cell cycle progression, and resistance to stress in immune progenitors and differentiated immune cells, as they do in many other cells types, consistent with a decline in overall immune function and proliferative capacity of the HSC pool with aging. FoxO transcription factors also regulate immune activity via specialized functions in different cell types. FoxO1 specifically regulates development and trafficking of B and T lymphocytes through several mechanisms, including survival and homing of pre-B cell and naive T cells in response to growth factor receptor regulation, pre-B cell maturation by induction of *Rag* genes and B cell receptor recombination, and B cell class-switch recombination and somatic hypermutation in response to germinal center formation. 

FoxO3 has been extensively studied in the immune system, and regulates not only lymphocyte function, but also innate immunity. Like FoxO1, FoxO3 regulates T and B lymphocyte survival and cell cycle progression, but FoxO3 additionally controls survival and entry of memory T cells into a quiescent state, critical for later response to infection. This particular function of FoxO3 is of great interest in the human immunodeficiency virus (HIV) field, as inhibitors of FoxO3 might be used to prolong survival of memory T cells during chronic HIV infection. FoxO3 also has similar roles in B cells, and is likely important for terminating immune responses to infection, and possibly for controlling lymphocyte responses that would result in autoimmunity. FoxO3 also has critical specific regulatory functions in innate immune cells: Controlling the number of neutrophils, monocyte/macrophages, dendritic cells (DCs), and erythrocyte progenitors (in opposition to erythropoietin signaling); directing neutrophil migration; and regulating inflammatory cytokine secretion by DCs in response to coinhibitory molecules [2,182]. Immune-specific functions of FoxO transcription factors, including the examples outlined here, are not well understood in the context of aging and increased metabolic stress, but may also contribute to the overall decline of cell-specific immunity observed in elderly individuals, including decreased maintenance of the HSC pool, increased susceptibility to infection, reactivation of latent viruses, and decreased immune surveillance with respect to cancer.

## 6. Mechanisms of Stem Cell Aging: Lessons from Transcriptional Reprogramming

In addition to their place on a spectrum of proliferative capacity, stem cells also exist on a spectrum of differentiation bounded by terminally differentiated unipotent effector cells at one extreme and pluripotent embryonic stem cells (ESCs) at the other [183]. Pluripotent cells, which can differentiate to any cell in the body, are thought to be extremely rare in adult mammals. Much work has investigated directed epigenetic manipulation of cell fate, inducing a cell to follow a completely different transcriptional program and as a result shift to an entirely different phenotype and spectrum of activity. 

The recent development of nuclear reprogramming methods used to generate iPS cells has created new opportunities for regenerative medicine using stem cells, but the mechanisms underpinning cell reprogramming remain incompletely understood, and many areas where stem cell manipulation can enhance regenerative medicine have yet to be explored [184,185]. Introduction of a cocktail of pluripotency-maintaining transcription factors, likely in combination with a series of stochastic epigenetic events, can direct terminally differentiated cells to revert to a state similar to that of an ESC [184,186,187,188,189], resulting in iPS cells that are pluripotent and germ-line competent, and exhibit the capacity for chimerism and teratoma formation and a gene expression profile characteristic of ESCs [190]. 

The exact nature of the pluripotency induction steps that take place during and subsequent to the expression of the exogenously transduced reprogramming transcription factors is an active area of research. Whether the two key “stemness” features of iPS cells, *i*.*e*., proliferative capacity and multi-lineage differentiation potency, arise from specific epigenetic events during the reprogramming process and result from the synergistic action of more than one of the reprogramming factors is unknown. Full reprogramming, *i*.*e*., the production of iPS cells, requires a minimal period of expression of these reprogramming transcription factors; this period was discovered by Yamanaka’s group to be significantly longer for induction of pluripotency in human cells compared to mouse cells, taking approximately three weeks [189]. Recent studies have also focused on the screening of small molecules capable of reprogramming [191,192], with one group achieving successful reprogramming using a combination of seven compounds [193]. 

### 6.1. Partial Reprogramming

However, iPS cells do not make ideal starting material for regenerative medicine or cell therapy [194]. Like ESCs, it is technically challenging to direct them to undergo exclusive differentiation along a specific cellular lineage, and they exhibit a shift in the self-renewal spectrum that confers a high risk of carcinogenesis, frequently forming tumors in animals [190,195]. It is possible that in the future we may be able to achieve partial reprogramming, resulting in the acquisition of renewed proliferative capacity and an increased differentiation lineage potential, but without other characteristics of ESCs and fully reprogrammed iPS cells, such as the capacity for chimerism and teratoma formation.

Partial reprogramming is the process of moving an adult cell on the spectrum of differentiation from limited multipotency toward pluripotency, without returning it to the completely pluripotent state of an ESC. Several groups are engaged in studying how partial reprogramming can most effectively be induced in adult stem cells, how it alters the transcriptional program and phenotype of adult stem cells, and how this approach may be used to preserve the potency, proliferative capacity, and regenerative utility of adult stem cells as they are cultured *in vitro* (reviewed in [196]). Observations from Yamanaka’s work in mouse cells suggests that selection based on expression of FBX15 yields partially reprogrammed iPS cells [197]. The definition of partial reprogramming described in that work was that partially reprogrammed iPS cells formed teratomas but lacked the ability to generate adult chimeric mice. 

A lesser degree of partial reprogramming has been described in umbilical cord blood cells cultured in medium supplemented with FGF4, SCF, and FLT-3 ligand [198]. These cells exhibited increased binding of acetylated histones H3 and H4 at the *OCT4* promoter and upregulation of OCT4 and Nanog expression, but their reprogramming was considered a partial event because they exhibited DNA hypermethylation in the *OCT4* gene region, and continued H3 and H4 acetylation at promoter regions for markers of terminal differentiation. Other studies have achieved partial reprogramming by administration of growth factors or transcription factors to redirect a non-pluripotent progenitor cell to a pluripotent phenotype [198,199,200]. Growth factor-induced partial reprogramming has been used to enhance plasticity in peripheral blood monocytes and subsequently to convert them to immature β endocrine cells [199]. During the observed limited life span of increased plasticity, these cells exhibited up-regulation of pluripotency markers. 

Recently some groups have undertaken to “directly reprogram” or transdifferentiate cells from one terminally differentiated phenotype to another using both developmental and lineage-specific transcription factors for therapeutic application in specific organ systems [201,202,203,204,205,206,207,208,209]. Partial reprogramming is a potentially promising approach to confer some of the desirable properties of ESCs onto adult stem cells or terminally differentiated effector cells, but it is evident that controlling partial reprogramming and resulting changes in potency requires a more complete understanding of underlying regulatory mechanisms. 

### 6.2. Molecular Mechanisms of Reprogramming

Discovery of mechanisms by which reprogramming events redefine the transcriptional program in adult cells, particularly signaling related not only to potency, but to telomere maintenance, oxidative stress, and senescence, will aid in generating techniques to increase the longevity of the adult stem cell in culture and preserve those cells *in vivo* [210,211,212,213,214,215]. Regulation of stem cell pluripotency and differentiation has been studied at the transcriptional and epigenetic level in ESCs, particularly mouse ESCs [216,217,218,219,220,221,222,223]. High-throughput sequencing methodologies are now used to characterize whole networks of regulation in ESCs [224,225] and analyze the roles of overlapping and interactive regulatory networks in determining stem cell fate, including the role of microRNAs [226] and epigenetic marks (reviewed in [227]). 

Regulatory networks in reprogrammed cells are also now being studied using genome-wide analytical tools, and initial results from studies of iPS cells derived from aged individuals suggests that reprogramming can undo many, though not all, effects of age (reviewed in [228]). SIRT1, critical for maintenance of stemness in multiple types of adult stem cells, is post-transcriptionally up-regulated during the reprogramming process [91]. Reprogramming of aged HSCs to iPS cells with subsequent re-derivation of HSCs showed comparable function to endogenous blastocyst-derived HSCs in marrow reconstitution assays [229]. Perhaps the most critical lesson regarding stem cell aging and loss of self-renewal gleaned from reprogramming research has been that cell aging as we know it is a largely reversible process, characterized not by permanent genetic mutations so much as progressive epigenetic inflexibility. 

## 7. Mechanisms of Stem Cell Aging: Lessons from Reprogramming Efficiency Studies

Studies of transcriptional reprogramming efficiency have proven very instructive in the area of methods for enhancing cell stemness and overcoming senescence. Several pathways controlling onset of cellular senescence must be differentially regulated to achieve reprogramming, including telomerase, p53, and mitochondrial/oxidative stress pathways [230]. Telomere length as a measure of cellular aging has revealed interesting differences between reprogrammed pluripotent cells and their embryonic counterparts. Many widely used human iPS cell lines derived from somatic cells display prematurely aged telomeres compared to hESCs with accompanying differential regulation of genes regulating telomere length. iPS cell clones derived from an hESC-derived mortal clone (for isogenic comparison) largely followed the same pattern with the exception of one clone spontaneously displaying levels of telomerase activity comparable to the parent hESC line, with maintenance of longer telomere length in culture [231]. From this finding we have learned that current reprogramming methods do not always result in iPS cells where the aging process has been fully reversed, but that further—likely stochastic—epigenetic events can enable full reversal of cell aging. 

Discovery of those specific events that result in maintenance of long telomeres is a relatively focused research problem that is likely solvable with the massive generation of transcriptional network data currently underway. Comparative studies of reprogramming in aged cells from multiple organs in mice have thus far demonstrated that age is an impediment to efficient reprogramming. However, many groups have successfully generated *bona fide* iPS cells from somatic cells of aged human subjects [228], and with ever-improving techniques have even demonstrated comparable reprogramming efficiency in fibroblasts from young *versus* old patients [232].

### Reprogramming Efficiency and Metabolic Stress

With respect to oxidative stress and mitochondrial function, the observation has been made that iPS cells rely on a Warburg-type switch to glycolytic metabolism. During reprogramming of fibroblasts to iPS cells, repression of H^+^-ATPase and up-regulation of the lipogenic enzymes acetyl-CoA carboxylase and fatty acid synthase is observed, as is the case in cells from many types of cancer, and inhibition of these lipogenic enzymes greatly decreases reprogramming efficiency [233]. Studies of mitochondria within human iPS cells have revealed that they revert to an immature state similar to those of an ESC, complete with reduced oxidative damage, contributing significantly to rejuvenation of the cell [228,230]. Pharmacologic induction of autophagy has also been shown to enhance reprogramming efficiency, perhaps through elimination of older, damaged mitochondria [88]. 

Metformin, an AMPK activator, has been shown to decrease reprogramming efficiency in multiple studies [119], despite the fact that AMPK activation induces endogenous antioxidant expression and reduces intracellular reactive oxygen species [234]. When activated, AMPK, which functions as a master sensor and regulator of intracellular changes in energy status, prevents transcriptional activation of *OCT4* (though not other reprogramming transcription factors) and prevents somatic cells from making the energetic switch to glycolysis, thereby effectively blocking reprogramming [119]. 

This is highly instructive for two reasons. First, the malignant component of teratomas derived from implanted iPS cells are driven by OCT4, and application of metformin to iPS cells (after reprogramming) has been used to suppress or block entirely the formation of iPS-derived teratomas [126], suggesting that this well characterized FDA-approved drug might enable clinical application of iPS cells without risk of carcinoma. 

Second, studies on the effects of metformin and other AMPK activators such as 5-aminoimidazole-4-carboxamide ribonucleotide (AICAR) on the reprogramming process have illuminated a critical path to achieving pluripotency: appropriation of energetic capital. It has long been known that many types of stem cells are able to survive in harsh, energetically unfavorable conditions such as hypoxia because of their ability to rely heavily on glycolysis (provided they are not calorically restricted), but the discovery that a particular metabolic phenotype is required for supporting the energetic requirements of the reprogramming process has resulted in the understanding that being able to readily shift to a glycolytic metabolic phenotype is a defining property of stem cells. The implications of these findings to the study of stem cells in aging are enormous, because manipulating the metabolic phenotype of a cell as a strategy to restore its function is an approachable problem.

Ascorbate, a potent antioxidant, has been shown to accelerate the kinetics of reprogramming and to alleviate cell senescence by reducing levels of p53 [235]. Curcumin, another antioxidant, has been shown to have similar effects on reprogramming efficiency [236]. It is possible that ascorbate enhances reprogramming in part through reduction of reactive oxygen species, but more likely by increasing the rate of transcriptome changes through other mechanisms: It is a cofactor for several enzymes, including collagen prolyl hydroxylases, HIF (hypoxia-inducible factor) prolyl hydroxylases, and histone demethylases [237], and may facilitate histone demethylation. 

Epigenetic modifiers, such as valproic acid, have been shown to enhance reprogramming efficiency, either alone or in combination with antioxidants. This further supports the idea that enabling histone demethylation confers epigenetic flexibility and enhances the ability of the cell to dramatically shift its transcriptional program [235]. Regulation of senescence and metabolic state through the mTOR hub seems to be of particular importance during reprogramming and is a pathway that can be fine-tuned to direct cell fate (reviewed in [238]). mTOR inhibitors, such as rapamycin and resveratrol (which is also a sirtuin activator), are known to slow cellular senescence in response to DNA damage by limiting the accumulation of p16 and p21, thereby enabling entry into a reversible quiescent state rather than an irreversible senescent state. These same compounds have been shown to increase the efficiency of reprogramming, in addition to other sirtuin activators, antioxidants, autophagy inducers, and PI(3)K inhibitors [236]. Interestingly, although it enhances reprogramming of normal somatic cells, resveratrol inhibits the stemness, epithelial-mesenchymal transition, and metabolic reprogramming of cancer stem cells to glycolysis through activation of p53, again highlighting the innate differences between the molecular circuitry of normal stem cells and cancer stem cells, a finding that can potentially be exploited for therapeutic purposes [239].

## 8. Therapeutic Approaches under Investigation

Given the growing evidence that many diseases of aging may reflect adult stem cell exhaustion, it is not surprising there is great interest in restoring adult stem cell function to ameliorate these conditions and regenerate aged tissues [23]. Adoptive transfer of fetal MSCs into adult mice has been shown to extend median lifespan of the animals [240]. Adult stem cell mobilization and transplant are two obvious strategies that have achieved moderate success for certain types of injury and disease in humans, and many types of adult stem cells have been utilized for this purpose [241]. MSC cellular therapy has proven to be safe for a number of vascular disorders, such as coronary artery disease, peripheral vascular disease, erectile dysfunction, and stroke, and is an attractive option for patients who are poor surgical candidates [242,243,244,245,246]. 

Despite these successes, the problem remains that adult stem cells from elderly donors, the very people who most frequently require enhanced peripheral stem cell function for tissue repair, undergo changes in their functional capacity as a result of aging (reviewed in [104]). This decline in functional capacity, therefore therapeutic utility, has been combatted using some surprisingly simple interventions: Conditioning with hypoxia prior to transplant, for example, has been extensively documented as effective for reducing reactive oxygen species production by adult stem cells and improving their therapeutic efficacy in many *in vivo* ischemia and other disease models [247,248,249]. This has proven sufficient to counteract the impaired oxidative stress resistance of MSCs from elderly donors [78]. 

Likewise, the use of naturally occurring antioxidant polyphenols, such as curcumin, has been documented to suppress inducible oxidative stress in human MSCs *ex vivo* and may prove to be a safe method for reducing oxidative damage to the *in vivo* MSC pool [250]. Rejuvenation of aged human MSCs has been achieved by seeding cell scaffolds with proangiogenic growth factors, resulting in improved functional capacity of the aged cells after implantation into an infarcted rat heart compared to aged cells seeded on untreated scaffolds [251]. Systemic administration of growth factors has also proven effective for restoring aged MSCs *in vivo*; in the case of senile osteoporosis, intraperitoneal injections of rhBMP2 were sufficient to reverse the osteoporotic phenotype, and this effect was mediated by an expanded MSC pool displaying increased proliferation and decreased apoptosis [252]. 

*Ex vivo* genetic modification has also been used to overexpress rejuvenating factors in aged bone marrow- and adipose-derived MSCs prior to therapeutic delivery. Transplantation of aged MSCs overexpressing telomerase and/or myocardin was more efficacious in stimulating arteriogenesis and blood flow in a limb ischemia model than transplantation of control aged MSCs [253]. A similar study achieved increased angiogenesis and less adverse matrix remodeling in a rat model of myocardial infarction using aged MSCs transfected with *TIMP3* or *VEGF* [254]. 

### 8.1. Transcriptional Reprogramming

The idea has been raised that it might be possible to exploit reprogramming techniques for renewal of the *in vivo* stem cell pool to combat diseases of aging [255]. While full reprogramming of stem cells *in vivo* to restore tissues degenerated as a result of age is not likely to manifest clinically until highly efficient reprogramming can be achieved through delivery mechanisms other than lentiviral vectors, the idea of “direct reprogramming” of cell fate in specific tissues *in vivo* has been pursued using developmental regulators that redirect a cell’s terminally differentiated state rather than returning the cell to a pluripotent state—what is known in the adult stem cell world as transdifferentiation, as opposed to dedifferentiation. This approach has been employed successfully to convert pancreatic exocrine cells to endocrine cells, rescuing the hyperglycemic phenotype in a mouse model of diabetes [256]. 

Generation of stem cells resistant to the phenotypic changes that accompany replicative senescence, such as arrested proliferation and decreased differentiation potential, would create a more ideal cell type for use in stem cell-based tissue engineering and cell therapy. *Ex vivo* reprogramming to achieve a kind of cell “reset” may in the future yield this improved cell source. iPS cells generated from HGPS patient fibroblasts display no evidence of progerin accumulation, nuclear envelope and epigenetic defects, or accelerated aging, suggesting this approach can in fact be used to reset an aged cell [58]. In the case of HGPS-iPS cells, differentiation results in the rapid accumulation of progerin and restoration of the accelerated aging phenotype [257]. 

However, this would not be an issue with physiologically aged donor cells. iPS cells derived from young and old non-progeroid human fibroblasts displayed no differences in mitotic activity after differentiation back to a fibroblast phenotype, suggesting that reprogramming is a successful approach to reset aged cells to a youthful phenotype in physiologically aged donors. In this study an excisable vector was used, further illustrating what might be a feasible approach to *ex vivo* rejuvenation of aged cells [232].

Stem cell rejuvenation techniques are also needed in situations where it is preferable to use cells from a specific donor who happens to be of advanced age, since HSC donor age is correlated with adverse events after infusion [258]. Meeting this need is critical for transplants with autologous or human leukocyte antigen (HLA)-matched sibling HSCs from elderly donors, which result in better outcomes in leukemia and lymphoma than HSCs from an HLA-matched unrelated younger donor [259]. Several groups have investigated the use of reprogramming transcription factors to restore differentiation potential and proliferative capacity of adult stem cells from aging donors. In one such study, Nanog was over-expressed in adult marrow-derived MSCs, resulting in reversal of lost myogenic differentiation potential and enhancement of proliferation comparable to that observed in neonatal marrow-derived MSCs [260]. It remains to be seen if other approaches to dedifferentiation will restore an unblemished phenotype to cells to the same degree that reprogramming appears to. 

### 8.2. Calorie Restriction and Pharmacologic Mimicry of Calorie Restriction

Calorie restriction as a therapeutic intervention to delay aging and extend lifespan has been extensively studied in animal models but, at levels that would confer significant clinical benefit, is unlikely to gain much traction due to low rates of adherence. Pharmacologic agents to reduce nutrient intake or absorption might be employed to this end (reviewed in [261]). The effects of diet and exercise to reduce body weight and correct metabolic disease on adult stem cell populations are unknown, although reduction in visceral fat has been shown to correct endocrine functions of adipocytes. Enhancement of PPARδ signaling has been suggested as an adjunct therapy to boost catabolism in visceral adipose tissue, perhaps in part through differentiation of adipose-resident MSCs to mitochondria-enriched small adipocytes [261]. To this end PPARδ agonists have been tested in clinical trials, but despite protective effects against obesity and diabetes, development was discontinued due to multi-organ cancer formation in animal models [262,263]. 

**Figure 4:**
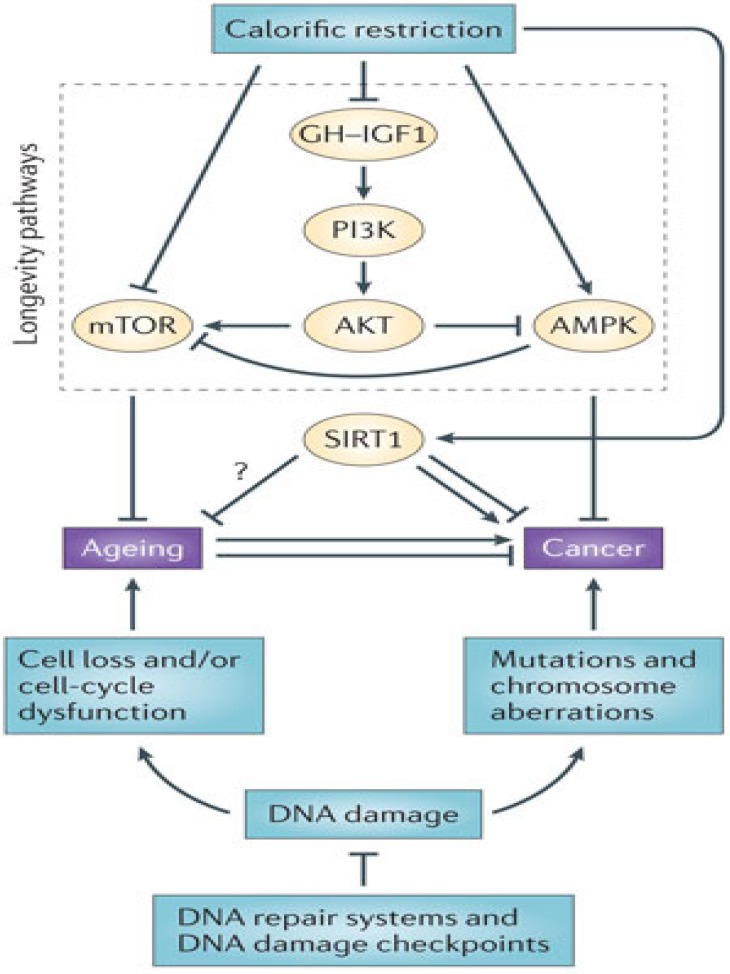
The growth hormone (GH)—insulin-like growth factor I (IGF1) pathway and its signaling cascade, which involves PI(3)K and Akt, can modulate longevity and cancer in model systems. Calorific restriction inhibits GH-IGF1 signaling and can also inhibit mTOR and activate AMP-activated protein kinase (AMPK) and sirtuin 1 (SIRT1). Interactions between components of these pathways, and with SIRT1, remain incompletely understood. The role of SIRT1 in modulating mammalian ageing has not been demonstrated, and it seems to have a dual role in cancer. DNA repair systems and DNA damage checkpoints prevent the DNA damage accumulation that contributes to cancer and ageing, although possibly through different cellular mechanisms. Reprinted by permission from Macmillan Publishers Ltd.: *Nature Reviews Cancer* [264], copyright 2013.

Other studies have investigated the use of pharmacologic agents to mimic the molecular benefits of calorie restriction for extending lifespan and healthspan (Figure 4, [264]). A recent report described extension of lifespan and healthspan in male mice with administration of metformin beginning in middle age [265]; past work has established this same phenomenon in invertebrates [266]. At the cellular level, treated mice displayed increased AMPK activity, decreased oxidative damage, and a transcriptomic shift mimicking the effects of calorie restriction. As a result the mice maintained sensitivity to insulin and low levels of systemic inflammation into old age. 

It should be noted that the dose of metformin used to achieve these effects resulted in serum drug levels an order of magnitude higher than what is typically achieved in patients when the drug is used as an antidiabetic therapy; a ten-fold higher dose proved toxic rather than beneficial in this study. As is the case for many pathways regulating longevity, cellular aging, and oncogenic resistance, the degree to which AMPK signaling is altered is likely to require a fine balance between too much and too little. This, combined with concerns about the pleiotropic effects of metformin *in vivo* which manifest differently with short-term *versus* long-term use, means that significant work is still needed before this potentially attractive therapy for systemic anti-aging can be safely employed.

### 8.3. Epigenetic Modification

The emerging field of translational epigenetics is aimed at correcting heritable but potentially reversible “epimutations” with chemical modifiers, and is gaining some traction in diseases of aging such as metabolic syndrome (reviewed in [267]) and cancer (reviewed in [268]) because it offers the possibility of targeting some of the same cell processes as transcriptional reprogramming without the associated risks of introducing exogenous genetic material. The treatment of epigenetically disrupted stem cells in cancer in particular may yield tremendous clinical benefit as the number of epigenetic modifiers grows, allowing for more specific targeting of known associated epimutations. 

In the area of metabolic disease, epigenetic modification with nucleic acids or small molecules may allow for extending the healthspan if not the lifespan of patients. The demethylase UTX-1, the activity of which increases in mid-life, removes gene inactivating marks such as histone H3 trimethylation on lysine 27 (H3K27me3) on members of the insulin/IGF-1 signaling pathway, enhancing their activity and resulting in decreased FoxO activity and age-related cellular decline. Restoration of H3K4me3 on an insulin-like receptor gene in *C*. *elegans* has been shown to decrease insulin/IGF-1 signaling, resetting the cell to a more naive epigenetic state and ultimately extending the life of the animal by 30% [139]. Although this effect was achieved through the use of RNA interference, the authors expressed optimism that in the near future small molecules might be employed to target epigenetic marks and/or modifying enzymes in a similarly specific strategy. 

Similarly, the histone methyltransferase SUV39H1 is protected from proteasomal degradation by enhanced binding to progerin in *Zmpste24^−^*^/*−*^ mice, resulting in increased H3K9me3 levels and compromised genome maintenance, which leads to accelerated senescence [269]. Targeting of SUV39H1 in this study resulted in amelioration of the progeroid phenotype in *Zmpste24^−^*^/*−*^ mice, including reduction of bone loss and extension of lifespan by 60%, suggesting a similar strategy might be useful in the context of normal aging. 

In an analogous approach, H4K16 hypoacetylation was targeted in the same mouse model to ameliorate the progeroid phenotype, with overexpression of the histone acetyltransferase Mof or addition of the histone deacetylase inhibitor sodium butyrate to drinking water promoting repair of damaged DNA and resulting in reduced evidence of disease [270]. Hypoacetylation of this mark was also found in aged wild-type mice, suggesting that aberrant histone acetylation may play a role in physiologic aging and administration of histone deacetylase (HDAC) inhibitors may have therapeutic value in disease of aging.

### 8.4. Strategies to Delay Senescence

Genetic modification strategies have specifically targeted known regulators of senescence and lifespan to combat diseases of aging. Preventing senescence, clearing senescent cells, or interfering with the senescence-associated secretory phenotype, in which cells release inflammatory mediators such as cytokines and matrix metalloproteinases, are all approaches that might lessen the contribution of cellular aging to chronic illness [271]. Given the complexity of the signaling crosstalk regulating senescence and associated events, identification of therapeutically targetable elements in this network—the “senectome”—is proceeding at multiple levels, the most recent of which includes senescence-associated micro-RNAs, which could be manipulated or used as clinical biomarkers [272]. 

In non-healing diabetic skin ulcers, siRNA- or *vivo*-Morpholino antisense-based gene therapy targeting of *CAV1* or *PTRF*, which are both turned on by oxidative stress in diabetic fibroblasts and induce p53-dependent premature senescence, inhibited senescence and accelerated ulcer repair [273]. Atherosclerosis, especially in the context of type 2 diabetes, is related to endothelial senescence and has been reduced using a variety of interventions targeting nitric oxide levels and bioavailability in the endothelial microenvironment, including eNOS gene therapy [274]. Gene therapy to induce telomerase activity in CD8 T cells, which undergo premature senescence in the context of HIV infection, results in enhanced proliferation and increased antiviral function [275]. While preliminary research in this area has not resulted in karyotypic changes or wildly altered growth kinetics of the CD8 compartment, the authors of this study emphasized the need for pharmacologic approaches that would mimic these effects without the need for *TERT* gene therapy due to the obvious risks. 

The use of pharmacologic agents to modulate senescence-associated pathways is a promising avenue to counteract the effects of aging in the clinic. Mice of both genders treated with rapamycin starting in mid to late life display extended lifespan and reduced incidence of cancer [276]. Treatment of cells from HGPS patients with rapamycin, results in enhanced clearance of the mutant protein progerin by autophagy and delayed onset of senescence [277]. Conversely, activation of Akt-mTOR signaling through inhibition of isoprenylcysteine carboxyl methyltransferase, the enzyme which processes prelamin A to lamin A and enables trafficking to the nuclear rim, in *Zmpste24^−^*^/*−*^ mice also delayed onset of senescence and improved disease phenotype. This suggests the Akt-mTOR axis, like the Wnt axis, is finely tuned and must be carefully manipulated to achieve therapeutic benefit [278]. Rapamycin is unlikely to be utilized extensively as an anti-aging therapeutic due to its side effects, which include hyperlipidemia and immunosuppression; however, newer analogs of rapamycin (rapalogs) are in development and may find use as anti-aging compounds, along with other agents that inhibit mTOR (reviewed in [279]). 

Pharmacologic activation of SIRT1 in a rat model of diabetes restored endothelial differentiation, pro-angiogenic chemokine secretion, and *in vivo* angiogenic activity of bone marrow-derived early outgrowth cells to that of cells from control animals [280]. Pharmacologic blockade of angiotensin II signaling through its type I receptor, which is used clinically to lower blood pressure and prevent insulin resistance in metabolic syndrome, also inhibits adipogenesis in adipose- and bone marrow-derived MSCs, both preventing further pathologic expansion of adipose tissue and helping to maintain an uncommitted progenitor pool for tissue homeostasis and repair [281]. In opposition to the enhanced production of 20-HETE by the cytochrome P450 system observed in metabolic syndrome, MSCs generate P450-derived epoxyeicosatrienoic acids (EETs) from arachidonic acids, and when administered exogenously these lipid mediators have been shown to decrease adipocyte differentiation of MSCs via an increase in heme oxygenase-1 and decrease in PPARγ, C/EBPα, and Fas and to reprogram adipocyte stem cells to a new phenotype displaying a smaller cell size, increased secretion of adiponectin, and decreased secretion of inflammatory cytokines [282]. EET agonists have also been shown to reverse a metabolic syndrome phenotype in an obese animal model, highlighting the therapeutic potential of targeting production of these molecules in the adult stem cell pool to combat this age-related disease phenotype in humans [283].

Given the complex and sometimes unpredictable nature of these emerging pharmaceutical and genetic approaches to age-related disease therapy, sometimes the simplest approaches to maintaining health are best. In the lifelong struggle between growth-promoting signaling pathways and stress resistance pathways, sleep has a critical place in determining the balance [171]. mTOR and FoxO signaling are turned on in distinct temporal windows during early and late sleep, respectively, in response to alterations in somatotrophic signaling, suggesting that a good night’s sleep truly does have restorative powers. Adult stem cells have been demonstrated to undergo significant circadian regulation in multiple studies, with HSCs, marrow- and adipose-derived MSCs, and cancer stem cells all subject to transcriptome modulation by core circadian regulatory proteins (reviewed in [284]). ESCs, in contrast to adult stem cells, are not subject to circadian regulation and have been shown to acquire molecular circadian oscillation upon differentiation; subsequent transcriptional reprogramming with *Sox2*, *Klf4*, *Oct3/4*, and *c-Myc* genes was shown to suppress circadian cycling, literally resetting the internal clock in the resulting iPS cells [285]. Interestingly, action of core circadian regulatory proteins on physiologic cellular processes is opposed by SIRT1 with aging (reviewed in [286]), and control of central circadian cycling by SIRT1 in the brain decays over time [287]. In general circadian rhythms break down with age, coinciding with the development of metabolic derangements and potentially contributing significantly to the organismal aging process (reviewed in [288]). Research into the impact of macro-environmental factors, such as organismal circadian rhythms, on stem cell niches may open new therapeutic avenues for manipulating stem cell fate in diseases of aging [289]. As therapeutic approaches go, good sleep hygiene is safe and free from side effects, cost-effective, and potentially contributes more than we currently know to the long-term maintenance of adult stem cell compartments.

## 9. Conclusions

Adult stem cells serve to replenish and direct repair at sites of tissue injury throughout the body, and exhaustion of dysfunction of an adult stem cell population *in vivo* with age results in degenerative disease. Several finely tuned and contextually regulated pathways coordinate the activities of tissue-resident adult stem cell pools over time in response to a host of cellular stressors in an effort to maintain the balance between growth-promoting function and oncogenic resistance. Manipulation of one or more of these pathways has the potential to prevent or reverse the impact of advancing age on adult stem cell function, but is fraught with the difficulty of tipping the balance toward metabolic derangement, or more likely toward cancer formation. Harvest and manipulation of adult stem cells *ex vivo* for use in regenerative medicine is a piecemeal approach to addressing systemic age-related chronic illnesses, but for now may prove to be a safer approach. In this regard, it is noteworthy that the clinical safety of HSCs and MSCs has been well documented, not in the least on the basis of decades of successful clinical outcomes of heterologous bone marrow transplantation. 

Further development of therapeutic approaches to maintain these cells *in vivo* requires that the mechanistic basis of their age-related degeneration or renewal be understood. This is an area continually being informed by studies of early-onset aging syndromes and of families exhibiting extreme longevity. Transcriptional reprogramming, which effectively wipes away all signs of age from most cell types, is also yielding valuable insights into what makes a cell young or old. Rejuvenating stem cells to stave off aging safely will require highly innovative approaches, but the results of this research will have far-reaching implications for regenerative medicine. 
